# Quality prevails over identity in the sexually selected vocalisations of an ageing mammal

**DOI:** 10.1186/1741-7007-8-35

**Published:** 2010-04-09

**Authors:** Elodie Briefer, Elisabetta Vannoni, Alan G McElligott

**Affiliations:** 1 Queen Mary University of London, School of Biological and Chemical Sciences, Mile End Road, London E1 4NS, UK; 2 Zoologisches Institut, Universität Zürich, Switzerland; 3 Current address: Anatomisches Institut, Universität Zürich, Winterthurerstrasse 190, 8057 Zürich, Switzerland

## Abstract

**Background:**

Male sexually selected vocalisations generally contain both individuality and quality cues that are crucial in intra- as well as inter-sexual communication. As individuality is a fixed feature whereas male phenotypic quality changes with age, individuality and quality cues may be subjected to different selection pressures over time. Individuality (for example, morphology of the vocal apparatus) and quality (for example, body size and dominance status) can both affect the vocal production mechanism, inducing the same components of vocalisations to convey both kinds of information. In this case, do quality-related changes to the acoustic structure of calls induce a modification of vocal cues to identity from year to year? We investigated this question in fallow deer (*Dama dama*), in which some acoustic parameters of vocalisations (groans) code for both individuality and quality.

**Results:**

We carried out a longitudinal analysis of groan individuality, examining the effects of age and dominance rank on the acoustic structure of groans of the same males recorded during consecutive years. We found both age- and rank-related changes to groans; the minimum values of the highest formant frequencies and the fundamental frequency increased with the age of males and they decreased when males became more dominant. Both age- and rank-related acoustic parameters contributed to individuality. Male quality changed with age, inducing a change in quality-related parameters and thus, a modification of vocal cues to male individuality between years.

**Conclusions:**

The encoding of individuality and quality information in the same components of vocalisations induces a tradeoff between these two kinds of signals over time. Fallow deer vocalisations are honest signals of quality that are not fixed over time but are modified dynamically according to male quality. As they are more reliable cues to quality than to individuality, they may not be used by conspecifics to recognize a given male from one year to another, but potentially used by both sexes to assess male quality during each breeding season.

## Background

Male vocalisations are a key component of sexual selection in many species. They can potentially provide receivers (both competitors and potential mates), with important characteristics about senders [1]. Two biologically significant characteristics of the caller that are likely to be encoded in sexually selected calls are individual identity and aspects of male quality [2-5].

Vocal cues to individuality generally result from inter-individual differences in the vocal production anatomy or in the way it is operated by individuals [6]. These cues are distinctive characteristics that allow senders to be recognised by their conspecifics [7,8]. Recognition plays a crucial role in a variety of social contexts. During the breeding season, males may benefit from signalling identity in the context of intrasexual communication through decreased aggressive competition over status in dominance hierarchies, and in the context of intersexual communication when being recognizable improves mating success [6,9,10].

Quality cues in vocalisations are usually a consequence of physical or physiological constraints that affect sound production [11]. Quality signals inform receivers about the overall phenotypic and genetic constitution of the sender [7]. Therefore, such signals can be used by males to broadcast their competitive abilities to rivals and females [1]. Mammal vocalisations, including human voice, have been shown to encode various indicators of quality such as body size, weight, age, dominance rank and fatigue [3,4,12-15].

For a given individual, identity is a fixed feature, whereas some of its phenotypic quality traits can change between years and even within years according to external or internal factors (life-history evolution) [16]. Therefore signals of individuality may be subjected to different selection pressures over time and have different properties compared to signals of quality [2,10]. Tradeoffs may exist between signals of identity that should be stable over time to allow individual recognition both within a year and between years, and signals of quality that should change as a function of the phenotypic quality of the individual in order to convey reliable information (honest signals). These tradeoffs could be partially resolved by using different features that are free to vary independently of one another to signal individuality and quality (segregation of information) [17].

According to the source-filter theory of voice production [18], mammal vocalisations are generated by vibrations of the vocal folds (*source*) and are subsequently filtered by the supralaryngeal vocal tract (*filter*). The source determines the fundamental frequency of the call (hereafter *F0*), whereas the filter shapes the source signal by selectively amplifying certain frequencies and dampening out others. This filtering mechanism produces peaks called *formants *[18,19]. The source and filter are usually independent processes as they can vary independently of each other [20]. F0 and formant frequencies can thus contain independent information and could be good candidates to encode identity and quality cues separately. However, acoustic cues to quality and individuality seem in several mammalian species to be linked to both source and filter characteristics [21,22].

When identity and quality cues are not conveyed in different and independent components of the vocalisations, quality-related changes to the acoustic structure of calls may induce modifications of the vocal cues to identity. We investigated this hypothesis in fallow deer, in which some acoustic parameters of groans code for both individuality and quality (body size and dominance status) [14,21].

The fallow deer (*Dama dama*) is a highly polygynous, size-dimorphic and long-lived species in which males only vocalise for a short discrete period each year. As they age, their phenotypic quality changes. They usually reach prime-age (in terms of mating) at six to seven years old, with survival and reproduction probabilities generally declining thereafter [23]. Groans are sexually selected calls mainly produced by mature males (≥ four years old). They are directed at other males, and are also important for mate attraction [24,25]. Fallow bucks groan only during the breeding season (late September to early November in the northern hemisphere), and are silent for the rest of the year. The breeding season corresponds to an intense period of competition amongst males for access to females and of vocal activity during which males may call at rates of more than 3,000 groans per hour. During this period, body condition of socially mature males declines due to increased energy expenditure and decreased foraging time [26]. Strong differences in phenotypic quality and mating success exist between males; those that invest more in display have higher mating and survival rates without losing more body condition compared to other males or suffering any long-term consequences (in terms of decreased survival or reduction in fecundity) [23,27].

We investigated if the vocal cues to male identity changed between years along with changes in dominance status and age. We first tested whether the phenotypic quality of males changed between years and the impact of these quality changes on mating success. We then examined age- and rank-related changes to the acoustic parameters of groans. Finally, we determined if these changes were related to modifications in the vocal cues to individuality. We predicted that if male quality changes between years, honest vocal quality traits should change accordingly. Such modifications should have important implications for signaling identity and quality in sexual selection.

## Methods

### Study site and population

The study was carried out on a herd of European fallow deer in Phoenix Park (53° 22' N, 6° 21' W), Dublin, Ireland. The age and identity of males used in this study were known because they had been tagged as fawns, as part of the management of the deer by the park authorities [28].

### Behavioural observations, mating success and dominance relationships

We conducted behavioural observations during the breeding season in 2000, 2002, 2003 and 2004. All-event recordings of agonistic interactions and matings were carried out every day from dawn to dusk (circa 11 hours per day). There were usually 7 to 13 observers in the field at all times and the coverage of animals was maximised. The measure of mating success for each male (years 2000, 2002 and 2003) was based on the number of observed copulations during the breeding season, which provides a very good estimator of reproductive success in fallow deer [29].

The outcomes of the agonistic interactions were used to calculate the dominance rank of each male by applying the David's score method [30]. This method is appropriate when interactions are recorded over a long period of time, because it takes into account repeated interactions between dyad members that may determine win/loss asymmetries [31]. Dominance ranks were calculated for males that interacted with at least 10% of other mature males. The rank value obtained for each male was then divided by the number of males present each year (55.5 ± 1.5, *N *= four years) to standardize this variable (*Rank*, one measure per male per year). The breeding season can be divided into two main periods according to the availability of mating opportunities: the prerut, which begins when all males have cleaned the velvet from their antlers and ends on the day before the first mating, and the rut, which is the period between the days on which the first and last matings occur [26]. Rank values available for males in 2002 and 2004 were prerut values whereas those available for males in 2000 and 2003 were rut values. Dominance ranks of fallow bucks can be measured soon after they regrow their antlers each year and before they become vocal. Male rank is thus well established before the rut so that prerut and rut rank values are highly correlated [26,32].

### Recordings and selection of groans

Recordings were made using a directional microphone (Sennheiser MKH-70; Old Lyme, CT, USA)connected to a digital audio tape recorder (Sony TCD-D100; Tokyo, Japan). Groans were recorded between dawn and sunset at a distance of 10 to 50 m from the vocalising animal. Vocalisations were imported into a computer using Avisoft-SASLab Pro v.4.38 (Avisoft Bioacoustics, Berlin, Germany) [33] at a sampling rate of 22.05 KHz and saved in WAV format, and at 16-bit amplitude resolution [34]. The recordings that did not contain energy above 8 KHz (as visualised on a spectrogram) were downsampled to 16 KHz for a better frequency resolution. Narrowband spectrograms of groans (FFT method, window length = 0.03 s, time step = 1,000, frequency step = 250, frequency resolution = 20 Hz, Gaussian window shape, dynamic range = 50 dB) were edited using Praat v.5.0.47 DSP Package (P. Boersma and D. Weenink, University of Amsterdam, The Netherlands) [35]. Vocalisations with high levels of background noise were not considered for analyses.

During the rut, fallow bucks feed very little, lose approximately 26% of their body weight, and the acoustic structure of groans is affected by fatigue [15,27]. We therefore analysed recordings taken between 9 October and 23 October when only a small proportion (15% or less) of the total number of matings had usually occurred, and when the majority of agonistic interactions among males were non-contact displacements [15,36]. For each male, selected groans had been recorded on the same day or on different days. Vocalisations produced within the same bout are more likely to be acoustically homogeneous than vocalisations emitted in different bouts [37]. To minimize this problem, the majority (84%) of the groans were randomly selected from different bouts and the remaining groans (16%) belonged to long vocalisation bouts but were never consecutive. We included in our analysis males between five and eight years of age because they had reached their maximum size and mass, and because this range of ages includes the males that are socially mature and account for the vast majority of vocalisations and matings [23,28,32].

### Acoustic analysis

**Figure 1:**
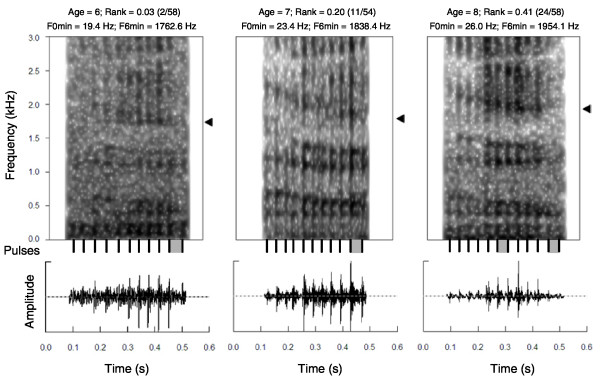
**Age- and rank-related changes in groans**. Spectrograms (above) and oscillograms (below) of three groans produced by the same male at six, seven and eight years old. Pulses are visible both on the spectrograms and oscillograms and are indicated as black vertical lines. Formants are visible as horizontal frequency bands on the spectrograms. Age-related changes to F0_min_, and rank-related changes to F6_min _are shown: F0_min _was measured for each groan as the inverse of the maximum interpulse interval (grey rectangle) on the oscillogram and increases with age (grey rectangle decreases); F6_min _is indicated with a black arrow and increases when the male becomes lower-ranking.

We analysed the vocalisations of 14 males recorded during either two (11 males) or three (3 males) breeding seasons (2000, 2002, 2003 and 2004). Groans are short and low-pitched vocalisations characterized by a pulse-train structure, which is visible on both the envelope and the spectrogram of the signal (Figure 1). The pulses represent the vibrations of the vocal folds and determine the fundamental frequency (F0) of the call. From the envelope of the call (amplitude vs. time), we measured mean, minimum and maximum F0 (F0_mean_, F0_min _and F0_max_), the number of pulses (Pulses), the duration of the groan (Duration) and a measure of cycle-to-cycle frequency variation (F0 perturbation), known as Jitter [19]. F0 is equivalent to the inverse of interpulse interval and this can be measured as the distance between consecutive pulse onsets [21]. Distances between pulses were measured using pitch analysis (Sound: To manipulation command) in Praat. We then calculated the values of F0 along the groan and averaged these values to obtain F0_mean_. Because all groans showed at least a modest frequency inflection, F0_min _and F0_max _were also included in the analyses. We also measured Pulses, and calculated Duration as the distance between the onset of the first pulse and the end of the last one. Finally, we quantified the variation of F0 along the call by calculating Jitter. Jitter has been used as a measure of voice quality in mammals [38], and in a similar manner to other source-related features, this parameter may contribute to vocal distinctiveness [21,39]. In our analysis, Jitter was calculated by dividing the average absolute difference between consecutive frequencies by the mean F0 per groan (peak-picking method) [40,41]. From the spectrum of the call (frequency vs. amplitude), we measured the frequency of maximum amplitude (Fr_max_) using peak detection with the package Seewave v.1.5.4 in R v.2.9.0 (R Development Core Team, Vienna, Austria) [42]. Fr_max _has been shown to signal individual identity in mammals [43-45].

Finally, the minimum frequencies of the first six formants (F1_min_-F6_min_) were estimated using Linear Predictive Coding analysis (LPC; Sound: To Formant (burg) command) in Praat. Formants are evident as horizontal frequency bands on the spectrogram of the groan (frequency vs. time and amplitude indicated as a colour scale; Figure 1). We compared the outputs of the LPC analysis with visual inspections of spectrograms to check if Praat was accurately tracking the formants. By performing a single LPC analysis on each groan, higher formants (F4 to F6) were better detected and therefore more accurately measured than lower formants (F1 to F3). We therefore conducted a double or a triple LPC analysis on each groan in order to get the best estimations of all formants. We first carried out an LPC analysis (time step = 0.01 s, maximum number of formants = three to five, maximum formant = 500 to 800 Hz, window length 0.04 to 0.07 s) to measure the frequencies of the first three formants (F1 to F3). Then we performed a second LPC analysis (time step = 0.01 s, maximum number of formants = four to seven maximum formant = 1,400 to 2,300 Hz, window length 0.04 to 0.07 s) to estimate the next three formant frequencies (F4 to F6). When the sixth formant was not detected by the second LPC analysis, we conducted a third LPC analysis (time step = 0.01 s, maximum number of formants = four to seven maximum formant = 1,900 to 2,300 Hz, window length 0.04 to 0.07 s). The decrease in formant frequencies along the groan reflects the elongation of the vocal tract occurring during vocalisations [46]. We calculated F1_min _to F6_min _from each groan by averaging the values over the last part of the call when formants become flat, after deleting spurious values and correcting for octave jumps. Minimum formant frequencies occur when the larynx is pulled down to the maximum extent, and therefore provide reliable information about the caller's body size [15,46]. We estimated the minimum spacing of the formants, known as minimum formant dispersion (Df_min_) [12] using the method described by [4].

### Statistical analyses

Data on dominance ranks and mating success were not available for all males whose vocalisations had been analysed. As a result, sample sizes (number of groans and number of males) varied among analyses including Age, Rank and Mating Success.

#### Age and dominance rank effects on mating success and on acoustic parameters of groans

We investigated the effect of Age on Rank and on Mating Success, the effect of Rank on Mating Success, and Age and Rank effects on acoustic parameters of groans, using Generalized Linear Mixed Models (GLMMs) fitted with Restricted Estimate Maximum Likelihood (REML, lme function in R) [47]. Based on scatterplots showing the relationship between the parameters used in the different GLMMs, we fitted as fixed effects linear, quadratic or log terms. To fit a quadratic term as a fixed effect, we z-transformed the variable and included all terms (linear and quadratic) in the model [48]. When more than one model was applied using different terms (linear, quadratic or log) to test a given relationship and both were significant, we assessed the best model based on the Akaike Information Criterion adjusted for small sample size (AICc) [14,47]. For a given model, the value of AICc is a measure of the information lost when the model is used to explain a particular pattern. Therefore, the model with the smallest AICc value is estimated to best fit the data set relative to other models considered [49]. In this case, only best models (lowest AICc) are presented in the results. Q-Q plots and scatterplots of the residuals of the dependent variables were inspected visually to ensure their normal distribution. The calculation of a coefficient of determination *R*^2 ^for GLMM is not obvious because of the presence of random effects. We thus estimated *R*^2 ^following [50] to describe the way models fitted the observed data, as follows: *R*^2 ^= 1 - exp (-2/*n *(*logL*_*M *_- *logL*_0_)), where *n *is the number of observations (groans), *logL*_*M *_is the standard log-likelihood of the model (which include fixed and random effects) and *logL*_0 _is the standard log-likelihood of the intercept-only model.

We carried out a first series of models to measure the effect of Age on Rank and on Mating Success, and the effect of Rank on Mating Success. In these models, male individual identity was fitted as a random term to control for repeated measurements of the same individual between years. In the models assessing the effect of Age on Rank and on Mating Success, linear (Age) and quadratic (Age^2^) terms were fitted as fixed effects (*N *= 12 individuals, 25 Rank values). In the model assessing the effect of Rank on Mating Success, log term (log(Rank)) was fitted as a fixed effect (*N *= 10 individuals, 16 Mating Success values). As the relationship between Age and Rank was significant, instead of including the original Mating Success values in the model testing the effect of Age on Mating Success, we included the residuals extracted from the model testing the effect of log(Rank) on Mating Success, which are thus independent of Rank. In the same way, in the model testing the effect of Rank on Mating Success, we included the residuals extracted from the model testing the effect of Age on Mating Success, which are thus independent of Age.

We then carried out a second series of models to examine Age- and Rank-related changes to the acoustic parameters of groans. In these GLMMs, year of recording nested within individual identity was fitted as a random term to control for repeated measurements of the same individual each year and across years. The date of recording (9 to 23 October) was included as a covariate (linear (Date) only or both linear (Date) and quadratic (Date^2^) terms) following [15] to control for individual acoustic variation over the breeding season. In the models assessing the effect of age on acoustic parameters, we fitted as fixed effects a linear term (Age) for Fr_max_, Pulses, Duration, and parameters related to F0 (F0_mean_, F0_min_, F0_max _and Jitter), and both linear (Age) and quadratic (Age^2^) terms for parameters related to formants (F1_min_-F6_min _and Df_min_; *N *= 305 groans, 9.84 ± 0.09 per individual per year, 14 individuals). In the models assessing the effect of Rank on acoustic parameters, a log term (log(Rank)) for Fr_max_, Pulses, Duration, and parameters related to F0 (F0_mean_, F0_min_, F0_max _and Jitter), and both linear (Rank) and quadratic (Rank^2^) terms for parameters related to formants were fitted as fixed effects (F1_min_-F6_min _and Df_min_; *N *= 245 groans, 9.80 ± 0.12 per individual per year, 12 individuals).

#### Between-years modification of the vocal cues to individuality

We quantified the individual distinctiveness of groans by performing a Principal Component Analysis (PCA) followed by a Multivariate Analysis Of Variance (MANOVA) and Discriminant Function Analyses (DFAs). We used a PCA carried out on all 2000, 2002, 2003 and 2004 recordings to eliminate redundancy due to the high intercorrelation of the acoustic variables in our data and to examine clustering among variables (*N *= 305 groans, 9.84 ± 0.09 groans per individual per year, 14 different individuals). We retained the Principal Components (PCs) of the PCA with eigenvalues greater than 1 (Kaiser's criterion). The scores of the five extracted PCs (PC1 to PC5) were confirmed for normality (Kolmogorov-Smirnov test) and used as input variables in the MANOVA and DFAs.

We used a MANOVA, with individual identity of bucks nested within year of recording fitted as a random term, to confirm within-year statistical differences in PC1 to PC5 scores across individuals. We then used DFAs with one factor (*individual*) to quantify the extent to which individuals can be classified on the basis of their groans and which group of variables (which PC) account most for this classification [51]. On the basis of the discriminant functions of the DFA, each PC score (corresponding to a groan) was assigned to the appropriate individual (correct assignment) or to another individual (incorrect assignment). We cross-validated our results by performing a leave-one-out classification, which is an appropriate method for small sample sizes [52-54]. We calculated the percentage of correct classification due to chance by applying a randomization procedure. The expected level of correct assignment was averaged from DFAs performed on 1,000 randomized permutations of the data set [54,55].

We first carried out a DFA and calculated percentages of correct classification on each year separately to assess within-year individual acoustic variation (same-year correct classification rate). As only one of the males recorded in 2000 was selected for the analyses, we carried out DFAs on PC1 to PC5 scores corresponding to 2002, 2003 and 2004 recordings (three different DFAs, *N *= 10.00 ± 1.00 individual per year).

To investigate between-years modification of vocal cues to male individuality, we then calculated percentages of correct classification of PC scores (corresponding to groans) of one year by a DFA carried out on a previous year (cross-year correct classification rate; function *predict *in R v.2.9.0) [56]. This procedure allowed us to calculate correct classification rates for groans recorded in 2003 and 2004 by projecting these data onto the discriminant functions generated by the 2002 DFA (*N *= seven and four individuals respectively), and for groans recorded in 2004 by projecting these data onto the discriminant functions generated by the 2003 DFA (*N *= eight individuals). Same-year correct classification rates (calculated using leave-one-out classification) and cross-year correct classification rates were then compared using a two-tailed exact permutation test (*N *= 13 individuals, 19 comparisons). A significant difference between same-year and cross-year correct classification rates would indicate a between-years modification of vocal cues to male individuality, according to the parameters measured in this study.

Statistical analyses were carried out using R v.2.9.0 (R Development Core Team, Vienna, Austria). All tests were two tailed and factors were considered to have a statistically significant influence if *P *< 0.05. All means are given with SEs.

## Results

### Age and dominance rank effects on mating success and acoustic parameters of groans

**Figure 2:**
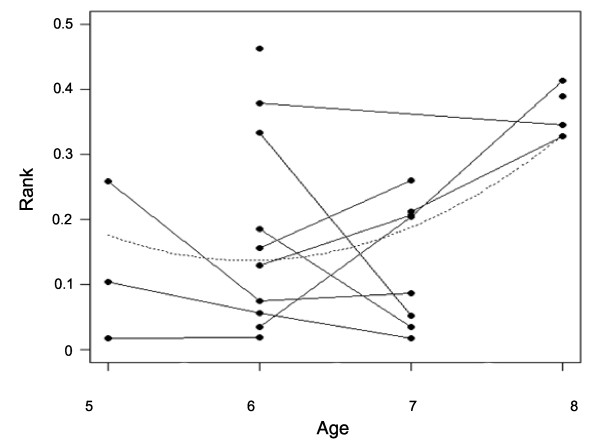
**Relationship between the age and dominance rank of males**. Lower values of dominance rank indicate higher-ranking males. Males tend to be higher-ranking at six and seven than at five and eight years old (quadratic relationship, *P *= 0.09). Dots represent mean values per year for each male and lines show repeated measures of the same individual across years.

There was a tendency for Age to affect Rank, with males being more dominant at six and seven years old than at five and eight years old (quadratic relationship, GLMM: *F*_1,11 _= 3.33, *P *= 0.09, *R*^2 ^= 0.23; Figure 2). Males tended to become higher-ranking by 0.14 ± 0.05 (for example, 8.12/58 rank positions) between five and six years old (or six and seven years old depending on the males considered) and to become lower-ranking by 0.12 ± 0.03 (for example, 6.96/58 rank positions) between six and seven years old (or seven and eight years old depending on the males considered). The effect of Age on Mating Success residuals (controlled for Rank) was not significant (GLMM: linear, *F*_1,4 _= 0.01, *P *= 0.93, *R*^2 ^= 0.0007). The log relationship between Rank and Mating Success residuals (controlled for Age) was significant (GLMM: *F*_1,4 _= 10.19, *P *= 0.03,, *R*^2 ^= 0.41), with males being more successful when high-ranking than when low-ranking. All males having a rank value ≤0.1 (for example, ≤5/58) obtained ≥14 matings. The most important difference was found for a male that obtained 46 matings when his rank was of 0.07 (4/54) and only six matings when his rank was of 0.26 (15/58). Therefore, male age influences their dominance rank, and higher-ranking males (six to seven years old) gain the most matings.

**Figure 3:**
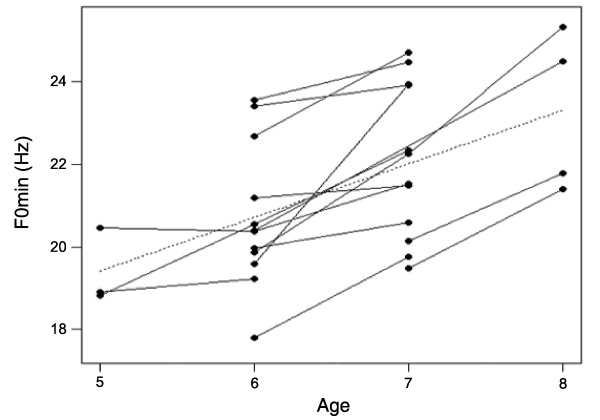
**Relationship between the age of males and the minimum fundamental frequency of groans**. The minimum fundamental frequency increases as males get older (linear relationship). Dots represent mean values per year for each male and lines show repeated measures of the same individual across years.

**Figure 4:**
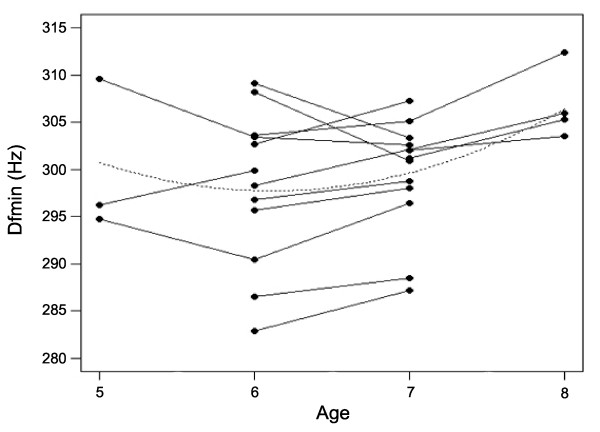
**Relationship between the age of males and the minimum formant dispersion of groans**. Males produce groans with higher minimum formant dispersion, indicating shorter apparent vocal tract lengths when they are five and eight years old than when they are seven and eight years old (quadratic relationship). Dots represent mean values per year for each male and lines show repeated measures of the same individual across years.

**Figure 5:**
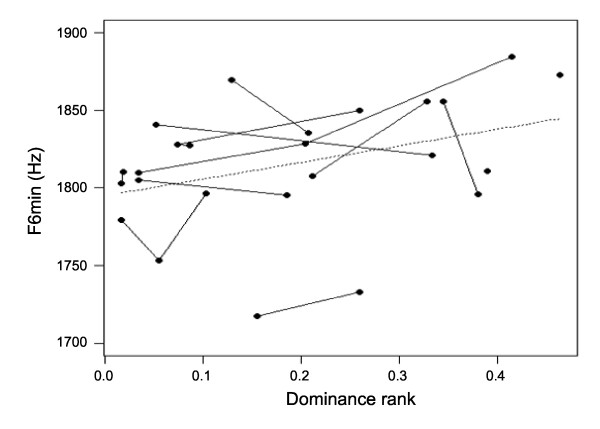
**Relationship between male rank and the minimum frequency of the sixth formant of groans**. Lower values of dominance rank indicate higher-ranking males. Males tend to produce groans with a lower minimum frequency of the sixth formant when higher-ranking (linear relationship, marginally significant, *P *= 0.05). Dots represent mean values per year for each male and lines show repeated measures of the same individual across years.

The results of the GLMMs investigating Age- and Rank-related changes to the acoustic parameters are shown in Table 1 (see also Additional files 1, 2 and 3). There was a significant positive linear relationship between Age and all measures of the F0 contour (F0_max_, F0_mean _and F0_min_), with males producing higher-pitched groans when older. This relationship was stronger for F0_min _(mean F0_min_: five years old, *N *= three, 19.39 ± 0.53 Hz; six years old, *N *= 12, 20.71 ± 0.50 Hz; seven years old, *N *= 12, 22.05 ± 0.54; eight years old, *N *= four, 23.26 ± 0.98; Figures 1 and 3). Age was also significantly related to F4_min_, F6_min _and Df_min_, with males having higher minimum formant frequencies and higher Df_min _(corresponding to shorter apparent vocal tracts) at five and eight years old than at six and seven years old (quadratic relationship, Figure 4). The effects of Age on Jitter, Duration, Pulses, Fr_max_, F1_min_-F3_min _and F5_min _were not significant (Table 1). Rank had a significant effect on F0_max_, with males having lower-pitched groans when higher-ranking. Rank also had a marginally significant effect on the highest formant frequencies (F5_min_, quadratic relationship, *P *= 0.07; F6_min_, linear relationship, *P *= 0.05), with males having lower minimum formant frequencies when higher-ranking (Figures 1 and 5). The effects of Rank on F0_mean_, F0_min_, Jitter, Duration, Pulses, Fr_max_, F1_min_-F4_min _and Df_min _were not significant (Table 1). Thus, the age and dominance ranks of males shape their groans, with both age and dominance rank affecting some measures of the fundamental frequency and the minimum frequencies of the highest formants.

**Table 1:** Age- and rank-related changes in the acoustic parameters of groans.

	Age					Rank				
		
Acoustic variable	Relationship	*df*	*F*	*P*	*R*^2^	Relationship	*df*	*F*	*P*	*R*^2^
**F0_max_**	**linear**	**1,16**	**6.85**	**0.02**	**0.13**	**log**	**1,12**	**6.15**	**0.03**	**0.15**
**F0_mean_**	**linear**	**1,16**	**10.90**	**0.005**	**0.23**	log	1,12	3.15	0.10	0.23
**F0_min_**	**linear**	**1,16**	**42.78**	**< 0.0001**	**0.30**	log	1,12	0.69	0.42	0.27
Jitter	linear	1,16	0.01	0.94	0.06	log	1,12	2.44	0.14	0.07
Duration	linear	1,16	0.54	0.47	0.21	log	1,12	0.04	0.85	0.23
Pulses	linear	1,16	2.30	0.15	0.18	log	1,12	0.82	0.38	0.18
Fr_max_	linear	1,16	2.34	0.15	0.12	log	1,12	2.02	0.18	0.17
F1_min_	linear	1,16	0.07	0.79	0.16	quadratic	1.11	1.14	0.31	0.13
F2_min_	linear	1,16	2.35	0.15	0.15	quadratic	1.11	0.21	0.65	0.16
F3_min_	linear	1,16	1.54	0.23	0.09	quadratic	1.11	1.27	0.28	0.06
**F4_min_**	**quadratic**	**1,15**	**5.82**	**0.03**	**0.26**	quadratic	1.11	3.27	0.10	0.25
**F5_min_**	quadratic	1,15	1.85	0.19	0.23	**quadratic**	**1.11**	**4.10**	**0.07**	**0.23**
**F6_min_**	**quadratic**	**1,15**	**15.40**	**0.001**	**0.38**	**linear**	**1,12**	**4.55**	**0.05**	**0.26**
**Df_min_**	**quadratic**	**1,15**	**8.09**	**0.01**	**0.37**	linear	1,12	3.16	0.10	0.30

Bold types indicate significant and marginally significant results.

Results of generalized linear mixed models to investigate variation in the measured acoustic variables, in relation to male age and dominance rank. Both age and dominance rank influence the F0 contour and the minimum frequencies of highest formants.

### Between-years modification of the vocal cues to individuality

The PCA performed on groans (*N *= 305 groans, *N *= 14 individuals, Table 2) generated five components (PC1 to PC5) that exceeded Kaiser's criterion (eigenvalues >1). These five components combined accounted for 77.5% of the variation in the original data set. Filter-related parameters were grouped in two different components that represent different aspects of the vocal tract function (PC1 and PC4); PC1 (*UpperF*) included higher formant frequencies (F3_min_-F6_min _and DF_min_), which are mainly related to the length of the vocal tract, whereas PC4 (*LowerF*) consisted of lower formant frequencies (F1_min_-F2_min_), which are generally associated with the shape of the vocal tract. Two components reflected aspects of source characteristics (PC2 and PC5); PC2 (*F0*) represented measures of the F0 contour (F0_max_, F0_mean _and F0_min_), whereas PC5 (*F0&Jitter*) included F0_min _and Jitter, a measure of voice quality based on the stability of vocal folds vibrations. Temporal parameters (Pulses and Duration) were highly correlated with PC3 (*TempP*). Finally, Fr_max _was not correlated with any of the five PCs. There was a significant within-year difference among males for principal component scores (MANOVA: *F*_30,274 _= 5.23, *P *< 0.0001). Thus, males of a given year produce groans that differ for five sets of correlated parameters, with each set of parameters reflecting a different component of the sound production apparatus.

**Table 2:** Factor loadings of the measured acoustic parameters on the first five principal components.

	Principal Component				
	
Acoustic variable	1	2	3	4	5
F0_max_	0.39	**0.84**	0.04	0.03	0.26
F0_mean_	0.35	**0.89**	-0.10	-0.12	-0.06
F0_min_	0.12	**0.68**	-0.23	-0.27	**-0.54**
Jitter	0.26	0.29	0.00	0.46	**0.74**
Duration	0.00	-0.22	**0.95**	-0.17	0.03
Pulses	0.21	0.32	**0.87**	-0.27	-0.05
Fr_max_	0.16	-0.05	0.05	0.27	-0.08
F1_min_	0.07	0.12	0.20	**0.64**	-0.27
F2_min_	-0.06	0.26	0.31	**0.57**	-0.25
F3_min_	**-0.51**	0.03	0.04	0.41	-0.31
F4_min_	**-0.80**	0.29	0.04	-0.15	0.21
F5_min_	**-0.92**	0.15	-0.01	-0.01	0.00
F6_min_	**-0.86**	0.26	0.05	-0.06	0.14
Df_min_	**-0.96**	0.26	0.05	0.03	0.05
Eigenvalue	1.96	1.59	1.37	1.19	1.11
Cum % variance	27.30	45.30	58.70	68.80	77.50

Bold types indicate the heaviest factor loadings (|*r*| >0.50). Eigenvalues and cumulative explained variances are given at the bottom of the table.

Correlated parameters of the calls are clustered into five principal components (eigenvalue >1) generated by a PCA carried out on the 2000, 2002, 2003 and 2004 recordings. Each principal component reflects a different constituent of the sound production apparatus

Each of the DFAs carried out on PC1 to PC5 scores corresponding to 2002, 2003 and 2004 recordings (*N *= 10.00 ± 1.00 individual per year) generated five discriminant functions. For each year, the first two discriminant functions, shown in Table 3, together accounted for 77.16 ± 0.03% of the variation (*N *= three DFAs). Both functions were highly correlated (|*r*| ≥0.5) with scores from PC1 (higher formants), PC2 (F0 contour) and PC3 (Pulses and Duration). The second discriminant function was also highly correlated with PC4 (lower formants) and PC5 (Jitter and F0_min_), but only for the 2004 DFA. The remaining three discriminant functions together accounted for 22.84 ± 0.03% of the variation (*N *= three DFAs). Each year, cross-validated DFA classified 38.66 ± 0.02% of groans to the correct male (same-year correct classification rate, *N *= three DFAs). This correct classification rate is higher than the percentage of correct classification due to chance (10.13 ± 0.01%; permutation test: 1,000 permutations, *P *= 0.0009 for each year). Cross-year correct classification rates of groans (PC scores) by DFAs carried out on the preceding year (22.43 ± 0.07%, correct classification rates of 2003 groans (*N *= seven individuals) and 2004 groans (*N *= eight individuals) by 2002 and 2003 DFA respectively) were still higher than chance level (9.81 ± 0.01%, permutation test: 1,000 permutations, 2003, *P *= 0.0009; 2004, *P *= 0.02). However, the cross-year correct classification rate of 2004 groans by 2002 DFA (15.0%, *N *= four individuals) was not significantly higher than chance level (11.09 ± 0.70%, permutation test: 1,000 permutations, *P *= 0.07). Same-year correct classification rates (36.09 ± 0.05%) were significantly higher than cross-year correct classification rates (21.62 ± 0.05%), indicating a between-years modification of vocal cues to male individuality (exact permutation test, *P *= 0.02). These results suggest that the vocal signals of male individual identity, which are parameters related to both source (larynx) and filter (vocal tract) characteristics, change by 13% (35 to 22% correct classification rates between consecutive years) between consecutive breeding seasons.

**Table 3:** Contribution of the acoustic parameters to the individuality of groans.

	Year of recording and discriminant function					
	
	2002		2003		2004	
	
PC	1	2	1	2	1	2
PC1 (HigherF)	**0.95**	0.01	**-0.59**	0.04	**-0.56**	-0.18
PC2 (F0 contour)	0.29	**-0.60**	0.44	**0.72**	**0.75**	-0.08
PC3 (TempP)	-0.10	**-0.78**	**0.66**	-0.37	-0.49	**0.69**
PC4 (lowerF)	-0.24	-0.16	0.38	0.31	-0.11	**0.58**
PC5 (F0&Jitter)	0.11	-0.17	0.31	-0.37	-0.48	**-0.64**
Cum % variance	0.43	0.73	0.53	0.76	0.60	0.82

Bold types indicate correlations greater than |*r*| = 0.5. Cumulative explained variances are given at the bottom of the table. See text for abbreviation of measured acoustic variables.

Correlations between the PC1 to PC5 scores and the first and second discriminant functions generated by DFAs conducted using the Principal Component scores corresponding to 2002, 2003 and 2004 recordings. PCs that are highly correlated with these two discriminant functions contribute the most to the individual identity of groans. Male vocal cues to identity are parameters determined by both vocal folds (PC2, PC3 and PC5) and vocal tract (PC1 and PC4) characteristics.

## Discussion

We investigated if the vocal cues to individuality of fallow bucks changed between years because of changes in phenotypic quality, using a longitudinal analysis with the same individuals compared at different ages. As males aged, their dominance ranks changed and rank was a good predictor of mating success. We found both age- and rank-related changes in the acoustic parameters of groans. All measures of the fundamental frequency contour (F0_max_, F0_mean _and F0_min_), the minimum frequencies of the highest formants (F4_min_, F6_min_) and the minimum formant dispersion (Df_min_) changed according to the age of males, with males producing groans with higher F0 and higher formant frequencies when older. Rank-related changes to the minimum frequency of the highest formants (F5_min _and F6_min_) were marginally significant, and F0_max _was also affected by rank changes, with males having lower F0_max _when higher-ranking. Within each breeding season, groans were individually distinctive, with higher formant frequencies (F3_min_-F6_min _and DF_min_), F0 contour (F0_max_, F0_mean _and F0_min_) and temporal parameters (Pulses and Duration) being the most important cues to male individual identity. We found that these signals of individuality substantially changed between breeding seasons. By including repeated measures and thus isolating the effects of differential mortality and age-specific signalling [57-59], our study is the first to investigate how both individuality and phenotypic quality shape vocalisations over time. Our results show that fallow deer vocalisations are honest signals of quality that are not fixed but are modified dynamically according to male phenotypic quality, showing a very robust example of 'truth in quality advertising' in animal communication [11].

Are fallow bucks using calls to encode their individuality or quality? Whereas the individuality of males is a fixed feature, we showed that one aspect of their phenotypic quality (dominance status) changed with age, and this had an impact on mating success (see also [23]). However, a comparison between the effects of age and dominance rank on vocalisations (Table 1) and the vocal cues to identity (Tables 3) shows that individuality and quality signals are partially conveyed in the same components of groans. According to the acoustic parameters measured in this study, calls were individualized within each breeding season, but only 38.7% (mean *N *per DFA = 10.0 individuals) of groans were assigned to the correct individual (see also [21], 36.6% and 53.6% with a stepwise procedure, *N *= 16 individuals). In fallow bucks, both source- and filter-related parameters can vary within individuals, F0 by the action of laryngeal muscles and formants by an elongation the vocal tract through retraction of the larynx [20,46]. This dynamic may result in acoustic parameters being unreliable cues to identity [38] and could explain the poor individuality of fallow buck groans that we found. However, further analyses would be needed to know if vocal cues to identity are present in other groan parameters (for example, shimmer, spectral slope, details of formant pattern, relative formant amplitudes) [38,60,61]. Indeed, Reby et al. [62] found a higher individuality of fallow buck groans than we did (87.9%) using a network classification based on the spectrum characteristics of calls. However, this higher correct classification rate could also be due to the lower number of individuals used in that study (*N *= four individuals) [62]. In the present study, we also found that the structure of groans changed between years according male quality, inducing a modification of vocal cues to identity. All these results suggest that groans are more reliable signals of quality than of individuality. There is some evidence that fallow deer females attend to male quality in choosing mates [63,64]. Because male quality changes from year to year, we suggest that females should attend to quality cues more than individuality cues to select the best quality males.

We found that F0 (especially F0_min_), the minimum frequencies of highest formants (F4_min _and F6_min_) and the minimum formant dispersion (DF_min_) changed as bucks got older, and these are therefore reliable cues to age. F0, which increased with age in our study, is negatively related to vocal fold length and positively related to vocal fold tension. The larynx is not constrained by neighbouring skeletal structures, so that vocal fold length is not particularly dependent on body size [12,20]. Moreover, males included in our study had reached their maximum size and mass, and groans selected for the analyses had been recorded early in the rut before males lost condition [15,27,32]. Therefore, the observed age-related changes to F0 cannot be explained by changes in body size.

In non-human mammals, age-related changes to vocalisations have been well studied during ontogeny [65-67], but studies investigating such changes occurring in adults as individuals get old are sparse. In baboons (*Papio cynocephalus ursinus*), Fischer et al. [3] found that males produced lower mean F0 when older. In red deer (*Cervus elaphus*), older males produce lower minimum and maximum F0 [4]. This decrease in F0 with age in red deer may be the consequence of the observed lengthening of the vocal folds throughout life, even after stags stop growing [5]. However, as we found an increase and not a decrease in F0 as males get older, this phenomenon probably does not occur in fallow deer.

The observed increase in F0 with age is more likely the result of decreased testosterone levels. In humans and closely related primates, the larynx is assumed to be a steroid receptor target organ [68-70]. Pubertal androgens cause thickening and lengthening of the vocal folds, and voice F0 is negatively related to circulating testosterone levels in adult humans [71-73]. Rolf and Fisher [74] found that testosterone levels in fallow deer peak during the rut for the first time in the fifth year of life. Moreover, the testosterone level for a seven-year-old male was lower than for a six-year-old male, suggesting a decrease in testosterone level after six years old, which could induce the increase in F0 observed in our study. An alternative hypothesis is that F0 may increase as a consequence of age-related anatomical and physiological changes in the larynx. Indeed, in humans, vocal folds atrophy, ossification of the laryngeal skeleton and increased stiffness of vocal fold tissue cause a rise in F0 with age in males [75,76].

We found that the minimum frequencies of the highest formants (F4_min _and F6_min_) and minimum formant dispersion (DF_min_) were higher when males were five and eight years old, than when they were six and seven years old. This indicates a shortening of the apparent vocal tract of males as they age. Higher formant frequencies and minimum formant dispersion are positively related to vocal tract length. Unlike the larynx, the vocal tract is constrained by the bones of the skull, jaw, and spinal column, so that its length strongly depends on body size. Thus, in mammals in which the larynx occupies a standard *high *position, formant frequencies are usually accurate cues to body size [12,77]. However, fallow bucks and red deer stags possess a descended larynx, which is also highly mobile. The larynx can be retracted during vocalisation by the sternothyroid and sternohyoid muscles, inducing an increase in vocal tract length and consequently a lowering of formant frequencies (fallow deer [46] and red deer [78]). In those species, only the minimum formant frequencies that an individual can produce when the larynx is fully retracted toward the sternum may be an accurate cue to body size (red deer [4], fallow deer [14]). In our study, because only mature males were included, the observed age-related changes in minimum frequencies of formants cannot be caused by body size changes with age. Furthermore, our results indicate an increase in formant frequencies with age, not a decrease that could result from an increase in body size with age.

The age-related changes in formant frequencies that we found are more likely caused by a combination of body condition differences in males, and senescence of the muscles responsible for laryngeal retraction. Unlike in fallow deer, humans, elephant seals (*Mirounga leonina*) and baboons produce calls with lower formants and lower formant dispersion when older [3,67,75], indicating vocal tract lengthening with age. In humans, this vocal tract lengthening has been suggested to be caused by lowering of the larynx in the neck as a result of stretching of ligaments and atrophy of the strap muscles of the neck [75].

We found that males tended to produce groans with lower minimum frequencies of the highest formants (F5_min _and F6_min_) when they were higher-ranking. Red deer stags have been shown to modulate vocal tract length and thus their formant frequencies as a function of the threat (body size) posed by opponents [79]. We suggest that a similar phenomenon exists in fallow deer, with bucks modulating vocalisations in relation to their dominance each breeding season. Fallow bucks use conditional competitive strategies to decide when to interact and fight, with fights being more likely to occur between opponents with similar dominance rank [36]. Furthermore, mature males increase and immature males (≤ three years old) decrease groaning rates following playbacks of groans from mature males [80]. These results strongly suggest that fallow bucks adjust their behaviour and vocalisations in response to the presence and quality of competitors. The dominance rank of males is established before they become vocal and remains largely stable across most of the breeding season [26]. Rank-related changes in the minimum values of the highest formant frequencies observed in our study may thus result from males adjusting their vocal tract length to their dominance status each year within the range of frequencies that each of them can physically produce. By doing so, males may reduce social costs associated with agonistic interactions, by avoiding contests with potentially stronger males (badge of status) [7,10]. This hypothesis is even more likely as, contrary to red deer that typically fully retract the larynx down to the sternum during roaring [78], fallow deer usually retract the larynx on average 52% during groaning [46], which could allow more variability in formant frequencies. Therefore, the formant frequencies of fallow buck groans may be even more prone to motivation- or hormone-related modifications than red deer formant frequencies.

F0 can be modulated through the action of the vocal muscles, thereby inducing changes in vocal fold tension [20]. The observed effect of rank-related changes to F0_max _of groans could thus result from the same mechanism as formants, with bucks modulating groan F0 according to their dominance rank, to avoid unmatched fights. Such F0 modulation occurs in humans, with men lowering or raising voice F0 when speaking to another individual who they perceive to be more or less physically dominant, respectively, to themselves [81]. Vannoni and McElligott [14] found a negative relationship between F0_min _and dominance rank by investigating the relationship between acoustic parameters and dominance rank for different individuals (cross-sectional method). We did not find that result in the present study and this is probably because we investigated this relationship using a more appropriate longitudinal method, in which groans of the same males were compared when they had different dominance rank values. These two kinds of methods may lead to slightly different results due to a potential confounding effect of differential mortality and age-specific signalling in cross-sectional studies [57,58,82].

Do all these observed age and rank-related changes induce a modification of vocal cues to the identity of bucks between successive breeding seasons? We found that both age-related parameters (F0 contour) and rank-related parameters (minimum frequencies of the highest formants) contributed strongly to the individuality of groans. Consequently, although groans were individualized within each breeding season, vocal cues to male individuality changed by on average 13% between consecutive years. These observed changes have a potential impact on individual recognition by conspecifics. Because males are silent outside the breeding season and live apart from females, individual recognition of a given male's groans by conspecifics may have to be re-established every year.

Females could benefit from mating with familiar males; familiarity with a given male being an indication of his investment in reproduction [8,26,83]. In fallow deer, long-term investment in vocal activity is related to mating success, suggesting that the familiarisation of females to male vocal characteristics is important for reproduction [26]. The present study suggests that this familiarisation may be more important within than between breeding seasons. During the breeding season, high-ranking males start groaning about three weeks before the first females are in oestrous [26] and this could be a strategy to familiarise females with the new sounds of their groans each year.

## Conclusions

To conclude, this study shows that individuality and quality signals can be encoded in the same components of vocalisations. This induces a tradeoff between these two kinds of signals over time, leading to one of them being more reliable than the other. Our results show that evolution favours phenotypic quality cues over individuality cues in fallow buck groans, with quality cues being more reliable than individuality cues over time.

## Abbreviations

AIC: Akaike Information Criterion; DFA: Discriminant Function Analysis; Df_min_: minimum Formant Dispersion; F0: Fundamental frequency; F0_mean_, F0_min _and F0_max_: mean, minimum and maximum Fundamental frequency respectively; F1_min_-F6_min_: minimum frequency of the first to sixth Formant; Fr_max_: Frequency of maximum amplitude; GLMM: Generalized Linear Mixed Model; LPC: Linear Predictive Coding; MANOVA: Multivariate Analysis Of Variance; PCA: Principal Component Analysis; PC1-PC5: Principal Component one to five; *R*: coefficient of determination; RELM: Restricted Estimate Maximum Likelihood; SE: Standard Error.

## Authors' contributions

EB analysed the data and wrote the manuscript. EV and AGM designed the study, collected the data and wrote the manuscript.

## Supplementary Material

Additional file 1 **Age: 6, rank: 2/58**. Groans produced by a male when he was six years old and high ranking (2/58). The fundamental frequency and minimum frequencies of the highest formants are low. Click here for file

Additional file 2 **Age: 7, rank: 11/58**. Groans produced by the same male (as in additional file 1) when he was seven years old and lower ranking (11/58). The fundamental frequency and minimum frequencies of the highest formants are higher than for the groans in Additional file 1. Click here for file

Additional file 3 **Age: 8, rank: 24/58**. Groans produced by the same male (as in Additional files 1 and 2) when he was eight years old and his rank had declined further (24/58). The fundamental frequency and minimum frequencies of the highest formants are higher than for the groans in Additional files 1 and 2. Click here for file
